# Revealing patterns of local species richness along environmental gradients with a novel network tool

**DOI:** 10.1038/srep11561

**Published:** 2015-06-25

**Authors:** Mara Baudena, Angel Sánchez, Co-Pierre Georg, Paloma Ruiz-Benito, Miguel Á. Rodríguez, Miguel A. Zavala, Max Rietkerk

**Affiliations:** 1 Copernicus Institute of Sustainable Development, Environmental Sciences Group, Utrecht University, P.O. Box 80115, 3508 TC Utrecht, The Netherlands; 2 Grupo Interdisciplinar de Sistemas Complejos (GISC), Departamento de Matemáticas, Universidad Carlos III de Madrid, Avenida de la Universidad 30, 28911 Leganés, Spain; 3 Instituto de Biocomputación y Física de Sistemas Complejos (BIFI), Universidad de Zaragoza, 50018 Zaragoza, Spain; 4 School of Economics and African Institute of Financial Markets and Risk Management, University of Cape Town, Private Bag X1, 7700 Rondebosch (Cape Town), South Africa; 5 Biological and Environmental Sciences, School of Natural Sciences, University of Stirling, FK9 4LA (Stirling), United Kingdom; 6 Forest Ecology and Restoration Group, Department of Life Sciences, University of Alcalá, Edificio de Ciencias, Campus Universitario, 28805 Alcalá de Henares (Madrid), Spain

## Abstract

How species richness relates to environmental gradients at large extents is commonly investigated aggregating local site data to coarser grains. However, such relationships often change with the grain of analysis, potentially hiding the local signal. Here we show that a novel network technique, the “method of reflections”, could unveil the relationships between species richness and climate without such drawbacks. We introduced a new index related to potential species richness, which revealed large scale patterns by including at the local community level information about species distribution throughout the dataset (i.e., the network). The method effectively removed noise, identifying how far site richness was from potential. When applying it to study woody species richness patterns in Spain, we observed that annual precipitation and mean annual temperature explained large parts of the variance of the newly defined species richness, highlighting that, at the local scale, communities in drier and warmer areas were potentially the species richest. Our method went far beyond what geographical upscaling of the data could unfold, and the insights obtained strongly suggested that it is a powerful instrument to detect key factors underlying species richness patterns, and that it could have numerous applications in ecology and other fields.

At large spatial extent, species richness is known to co-vary with climate, typically increasing from the poles towards the tropics^1^,^2^,^3^,^4^. Climate determines water availability and ambient energy levels, although the mechanisms connecting these factors to species richness and diversity are still debated^1^,^5^,^6^,^7^. Usually plant richness patterns correlate strongly and positively with water availability, and e.g. tree richness patterns have been found to be strongly associated with rainfall in both the tropics^2^ and the extratropics^5^,^8^, while ambient energy becomes limiting in cold climates^1^.

Analyses of species richness-climate relationships at large geographical extents often use data at coarse grains (e.g. square cells of 10 × 10 km or larger), obtained from scaling up local site data that are supposed to be too noisy to reveal climatic and environmental signals (e.g. refs. 4,9). However, various broad-scale, grid-based studies show that different grains can lead e.g. to changes in the steepness (or even in the direction) of the relationship between species richness and the explanatory climatic variables (see e.g. refs. 10, 11, 12, 13, 14, 15, 16, 17, 18). Similarly, scaling up local site data to larger cells is likely to wipe out local information, related e.g. to environmental variation, or to the community structure (i.e. changing the relative ubiquity of species, and introducing species co-occurrences that do not exist at the community level). This in turn would prevent detecting how variations in local species richness relate to climate over broad spatial extents.

Here we study the patterns of species richness at large extent using data at small, local, site scale, with the help of network analysis, which allows to include information from the whole network at the local level. Networks in ecology have been used mainly to describe food-webs or plant-pollinator interactions (see e.g. refs. 19, 20, 21, 22). Some works also used network approaches within a trophic level (e.g. refs. 23, 24, 25, 26). In food-webs and pollinator-plant webs, the species interactions directly define their networks. When representing species within a trophic level, the network links instead often represent co-occurrence, as built out of a classical presence/absence matrix, or in some cases a matrix of species abundances. This kind of matrices have been largely investigated in ecology, e.g. to explain geographical species patterns empirically (e.g. ref. 27), to identify species associations (e.g. ref. 28) or to study theoretically the relationships between patterns of species richness and range sizes^29^. Network techniques are powerful also when applied within a trophic level, because they account for all the species co-occurrence throughout a certain area in a synthetic way, which allows for global description and insights, without losing the local information.

In the present work, we introduce into ecology a new network technique, called the “method of reflections”, which has been used in economics to show that a few factors drive the product export of countries^30^. We adapt this method to analyse the distribution and richness of species, obtaining a technique that summarizes information on community structure and species distribution throughout the entire dataset, and that uses this information to get an indication of potential species richness at the local level. The method of reflections introduces a new way of removing noise from local site species richness, identifying to which extent species richness of each site is further away (or closer to) its potential.

To demonstrate the added value of this method, we consider the case of woody plants (i.e. trees and shrubs) in mainland Spain, using data from the Spanish Forest Inventory (SFI), which describes forests at local, site scale (i.e. circular plots of ≤25 m radius). Woody plant species richness in this area has been associated to major climate characteristics, namely mean annual temperature and annual precipitation, in contrasting ways, depending whether the studies use local site data^31^, or large cells^32^. While no study, to our knowledge, has found a clear pattern for local species richness in Spanish forests using a dataset as extensive as the SFI, Terradas *et al.*^31^ analyse a small subset of community level data sampled in canonical (i.e. well-preserved) forest stands (thus with minimum heterogeneity due to management practices). They detect an increase of species richness with mean annual temperature, and a decrease with annual precipitation. Although their study sites are located in NE Spain, this result can be expected to be roughly indicative of the situation across peninsular Spain, since the study covered a strong climatic gradient, encompassing a range of woodland types representative of major Mediterranean and Atlantic woodland formations in Spain. Conversely, the grid-based analysis by Vetaas & Ferrer-Castán^32^ for the Iberian Peninsula finds opposite trends using cells with a resolution of 50 km, i.e., coarse grain: woody species richness increasing with annual precipitation, and decreasing with mean annual temperature. It is not clear why opposite results are obtained for species richness at the local and coarse-grain scales, but it cannot be excluded they are related to the above mentioned sensitivity of upscaling (grid-based) techniques to underlying heterogeneity or grain size (e.g. refs. 13,33).

The objective of this work is twofold. Firstly, we aim at demonstrating the effectiveness of the method of reflections in removing noise and isolating the signal of potential species richness, calculated at the site level from an ecological presence/absence dataset. Secondly, we apply the method of reflections to investigate how the variations of woody plant species richness across local communities in Spain relate to major climate characteristics. For a comparison, we also explore these relationships with the classical upscaling analysis. As we will show, after removing the noise in our data with the method of reflections, we effectively detect climate relationships with local woody species richness, specifically an increase with mean annual temperature and a decrease with annual precipitation, thus displaying similar relationships to those observed in canonical sites (i.e. well-preserved local communities^31^), and in contrast with the opposite patterns that emerge from the upscaling analysis (as in ref. 32).

In the following two sections, we introduce the method of reflections, and we illustrate its validity and potential with an example of its application to synthetic datasets. We then apply the method to analyse the SFI, and discuss these results and the method relevance and wide applicability. Finally, we report the descriptions of the SFI dataset and of the analyses.

## The method of reflections

Here we introduce the “method of reflections”^30^, which we employ to analyse species richness. We briefly mention also how the method applies to the species frequency of occurrence, which is equivalent to species range size for those species whose range distribution is limited to our study area. We consider the species and the sites as separate parts of a so-called “bipartite” network^34^, in which plants are connected to the sites where they grow.

For the species used in the analysis, we introduce the species presence/absence matrix *M*, whose elements *M*_*ps*_ are equal to 1 if a plant species *p* is present in a site *s*, and 0 otherwise. The matrix *M* represents a bipartite network with two kinds of nodes, species and sites, where each species is connected to the sites where it is observed. The method of reflections introduces a family of variables (hereafter referred to as “reflections”) that characterize the species and the sites, producing a symmetric set of measures. We start from the definition of two widely used ecological quantities, species frequency of occurrence and site species richness:


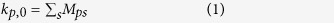



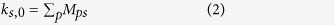


where the zeroth order reflection *k*_*p,0*_ is the number of sites where the plant species *p* is observed (i.e. its frequency of occurrence), and the zeroth order reflection *k*_*s,0*_ represents the number of species per site *s* (i.e. site species richness). These reflections correspond to the so-called “node degree” of species and sites, respectively (e.g. ref. 35).

The method of reflections introduces a generalization of these two quantities, defining *N* reflections for each type of node, i.e. for both the species and the sites. The reflections of sites (*k*_*p,N*_) and species (*k*_*s,N*_) are calculated iteratively as the average value of the previous level properties:


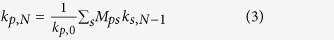



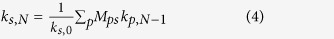


for *N* > 0, thus obtaining a set of reflections (*k*_*p,0*_, *k*_*p,1*_, *k*_*p,2*_, … *k*_*p,N*_) for the plant species and a set (*k*_*s,0*_, *k*_*s,1*_, *k*_*s,2*_, *… k*_*s,N*_) for the sites. For example, the first-order species reflection, *k*_*p,1*_ is defined as:


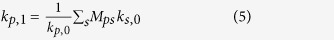


and it is thus the average species richness of the sites where a plant species *p* was observed. The first site reflection, *k*_*s,1*_ is defined as:


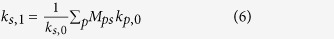


i.e. the average frequency of occurrence of the species growing in a site *s*. These first order reflections have been occasionally used in previous studies^29^,^36^.

**Figure 1:**
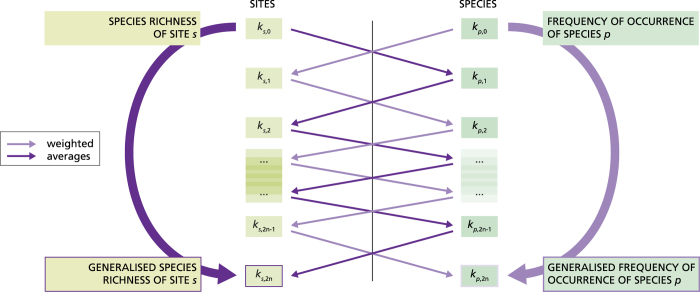
Schematic of the repeated averaging in the method of reflections. The reflections are calculated as repeated averages, represented by the straight arrows connecting the sites (left) and species (right) reflections. Dark arrows connect reflections related to species richness; light arrows connect reflections related to frequency of occurrence. Each reflection of a given order and type of node is the weighted average of the reflections of the previous order and the other type of node. Thus, the information travels through the reflections as symbolised by the straight arrows, so that for each type of node, high order, even reflections contain the same information of the zeroth order reflections of the same node type, as symbolised by the (curved arrows). Namely, *k*_*s,2n*_ and *k*_*p,2n*_ are related to *k*_*s,*0_ and *k*_*p,*0_, respectively.

The method of reflections continues the iteration process to higher orders, and thus the following iteration for the sites introduces *k*_*s,2*_ as the weighted average species richness of a site *s*, where the species assume different weights according to their *k*_*p,1*_, i.e. to the species richness of the sites where they were observed (see Fig. 1). Species living on average in species-richer sites (i.e. species that are more “social”) have larger weights. In other words, *k*_*s,2*_ is an index related to site species richness, which includes information on the species richness of all the sites where the species in site *s* are observed. The process is analogous for the species reflection *k*_*p,2*_. Iterating further, as shown above for the sequence *k*_*s,0*_ *– k*_*p,1*_ *- k*_*s,2*_ , the information travels between the even reflections of one type of node and the odd reflections of the other type^37^ (see an illustration in Fig. 1). Thus, we obtain two families of reflections for each type of nodes. For sites, even reflections (with order larger than zero, i.e. *k*_*s,2*_, *k*_*s,4*_,*…*) represent generalized measures of species richness, and are thus related to the site zeroth order reflection *k*_*s,0*_, while odd reflections (i.e. *k*_*s,1*_, *k*_*s,3*_, *k*_*s,5*_,*…*) are generalized average measures of the frequencies of occurrence of the species present at the site, and thus are related to *k*_*p,0*_ (the zeroth order reflection of the species). For each plant species, even reflections (with order larger than zero, i.e. *k*_*p,2*_, *k*_*p,4*_,*…*) represent generalized measures of its frequency of occurrence, whereas odd reflections (i.e. *k*_*p,1*_, *k*_*p,3*_, *k*_*p,5*_,*…*) are related to the species richness of the sites where the species was observed (Fig. 1; see also Table 1 for a summary of the reflection meaning).

As an example, we consider high order, even site reflections (e.g. *k*_*s,18*_), which represent generalized site species richness. Let us imagine a site that has many species because it is at an early successional stage as a consequence of a “disturbance”, but most of its species tend to live in monospecific stands, or with few other species, in the rest of the dataset. The generalized species richness of this site would then have a relatively low value, because most of its species get low weights. Analogously, if a site is species poor, but its few species generally co-exist with many other species in the rest of the dataset, the site generalized species richness would be high, because the “social” species have large weights. Thus, generalised species richness is an indication of the potential species richness of a site, keeping into account the general properties of the species that compose its local community, as estimated from the whole network (i.e. the dataset). In the following section we illustrate this mathematical property with an example.

Finally, we notice here that when stating that the reflections are “generalized” measures, we imply that the reflections smooth out the variables they represent throughout similar nodes (i.e. sites or plant species) in the network. In other words, the method isolates the signal from the noise. It is important to note that the generalized measures are not new estimations of site species richness and species frequency of occurrence, but they rather are new, smoothed indices that contain averaged information about those variables. The iterative procedure used to calculate them stabilizes the averaging effect, and in fact, the reflections tend to converge to similar values when *N* is large^37^. While their values are not informative per se (and as such the reflections can be considered as indices), yet they contain a lot of information in their tiny deviations^30^, as a consequence of the inclusion at node level of information from the whole network, which leads to the noise removal.

### An illustrative application to synthetic datasets

Here we illustrate with an example that the method of reflections can identify the signal of (potential) species richness even when it is hidden behind the randomness of a noisy dataset. We generated two synthetic datasets where the species are stochastically distributed, each according to a known probability of occurrence as a function of the environmental conditions in the sites. In the first dataset (D1), the number of species that could potentially occur in a site changed along the environmental range. In the second dataset (D2), the number of species that could potentially occur did not change along the climatic gradient. We used this dataset to check that the method did not find spurious correlations.

For both datasets, we generated large matrices of species presence/absence in sites (110 species, 10000 sites). To keep the example simple, the environment in each site was described by only one variable, namely annual precipitation, which was randomly assigned to the site. The annual precipitation varied between 0 and 1500 mm y^−1^, and to obtain a uniform distribution of the values across the sites, it was sampled every 25 mm y^−1^. The probability of occurrence of each species varied with the annual precipitation, and the distributions were normal, truncated between 0 and 1500 mm y^−1^ (see Supplementary Note, Fig. S1). In the dataset D1, we assumed that several species had identical probabilities of occurrence, and the number of species that preferred one end of the precipitation gradient was higher than the number of species that preferred the other end of the gradient. In detail, the number of species that had the maximum probability at a certain precipitation value decreased linearly with precipitation itself, with 20 species having a high probability of occurrence in the driest areas (with mean of the probability distribution μ = 125 mm y^−1^ and standard deviation σ = 100 mm y^−1^), 10 species in the wettest (μ = 1375 mm y^−1^ and σ = 100 mm y^−1^), and with the number of species decreasing linearly in between these two extremes. We included also twenty “generalist” species that had a broad probability distribution all over the precipitation range (μ = 750 mm y^−1^ and σ = 500 mm y^−1^, see Supplementary Note, Fig. S1). In the second dataset (D2), we did not include any trend in the number of species that preferred a certain precipitation. We generated 110 species with mean value of the probability of occurrence chosen at random between 0 and 1500 mm y^−1^ (sampled every 100 mm y^−1^ to sample uniformly) and standard deviation of 900 mm y^−1^. For both datasets, the species were then assigned randomly to the different sites, with a probability that was calculated as a function of the site annual precipitation. For each site and species we generated a random number between 0 and 1, and we assigned the species to the site only if the random number obtained was smaller than the probability of occurrence of the species in that site.

**Figure 2:**
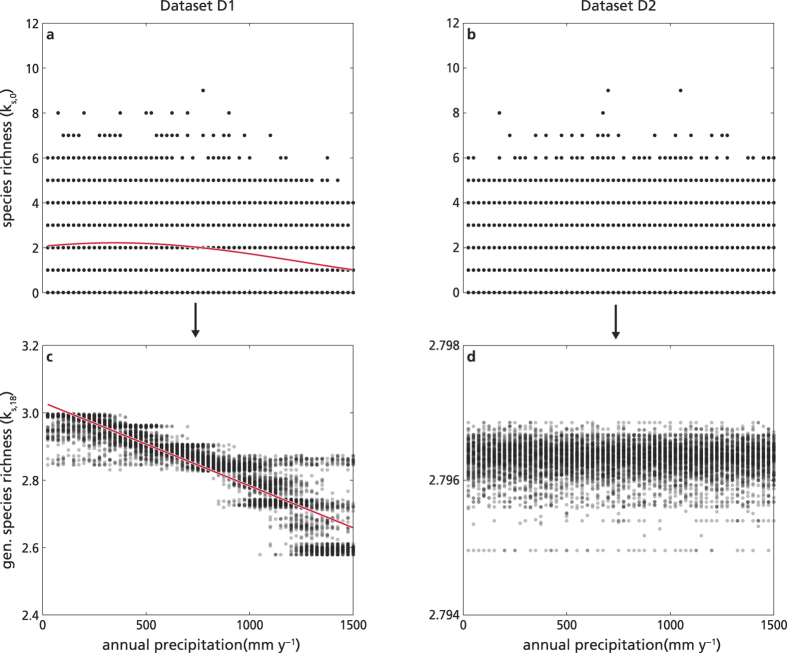
The method of reflections illustrated with synthetic, stochastic datasets. We use two datasets where the species distribution, respectively: (**a**,**c**) follows the precipitation gradient (dataset D1); (**b**,**d**) does not include any trend with precipitation (dataset D2). Site species richness (*k*_*s,0*_, panels **a**,**b**) and generalised species richness (*k*_*s,18*_, panels **c**,**d**) as a function of annual precipitation (mm y^**−**1^). For dataset D1, **a**) species richness decreases as a quadratic function of annual precipitation (with small variance explained, R^2^ = 0.07), while **c**) generalized species richness decreases linearly with annual precipitation (with large variance explained by the model, R^2^ = 0.84). For dataset D2, no significant trend is observed neither in species richness (**b**) nor in generalized species richness (**d**)(all model fits have R^2^ < 0.01). See Supplementary Note, Table S1 for details on model selection.

We started from dataset D1 and plotted the species richness data as a function of the annual precipitation of the sites (Fig. 2a). The randomness in the process of assigning the species to the sites hid the linear trend in the species that could potentially live along the precipitation gradient. The best model fit (obtained with Generalised Linear Models – GLM – and selected as described in detail below) was a quadratic model, with species richness decreasing with annual precipitation as expected, but explaining very little variance (R^2^ = 0.06, Fig. 2a and Supplementary Note, Table S1). We then applied the method of reflections up to the order *N* = 18, and we plotted the generalised species richness *k*_s,18_ as a function of the site annual precipitation (Fig. 2c). The GLM analysis showed that the best fit supported a linear decrease of generalised species richness with annual precipitation, explaining more than 80% of the variance (R^2^ = 0.84, Fig. 2c and Supplementary Note, Table S1). To verify that the method did not introduce spurious correlations, we performed two different tests. Firstly, we bootstrapped the dataset D1, maintaining the same matrix of species presence/absence but shuffling around the site precipitation values randomly. We repeated the shuffling *n* = 1000 times, obtaining *n* datasets to which we could apply the method of reflections. No correlation was observed between the generalised species richness and the annual precipitation in these *n* datasets (average R^2^ ~ 0). Secondly, we applied the method of reflections to the dataset D2. As expected, no model was supported for the fit of annual precipitation and species richness (Fig 2b), but most importantly, also no model fit was supported for generalised species richness *k*_*s*,18_ and precipitation (Fig 2d). We therefore can state that the method of reflections removes the noise in a large dataset of species presence/absence, identifying the potential species richness signal using only the species composition of the site and their assembly pattern throughout the dataset. With the last two examples, we also verified that the method does not introduce artefactual patterns but it simply highlights hidden signals.

Finally, with the synthetic dataset D1, we illustrated two other important features of the method (see Supplementary Note). Firstly, we showed that the generalised species richness (*k*_s,18_) was a measure of the potential richness of the site, obtained including information about the species patterns of co-occurrence throughout the dataset at site level. This proof was possible for this dataset, because we could estimate the potential richness from the theoretical probability of occurrence of each species in each site (see Supplementary Note, Fig. S2), and it confirmed our explanation of the method as derived from the mathematical formulas (Eqs. 3, 4 and Table 1). Secondly, we could show that the method could identify how far (or close) the sites were from their potential richness, using the changes in site ranking from generalised species richness *k*_*s*,18_ to species richness *k*_*s*,0_ (see Supplementary Note, Fig. S3).

### Patterns of species richness in the Spanish forests

In this section, we illustrate the results obtained applying the method of reflections to study the patterns of woody species richness along a climate gradient in Spain, using the Spanish Forest Inventory dataset (see below for a description).

**Figure 3:**
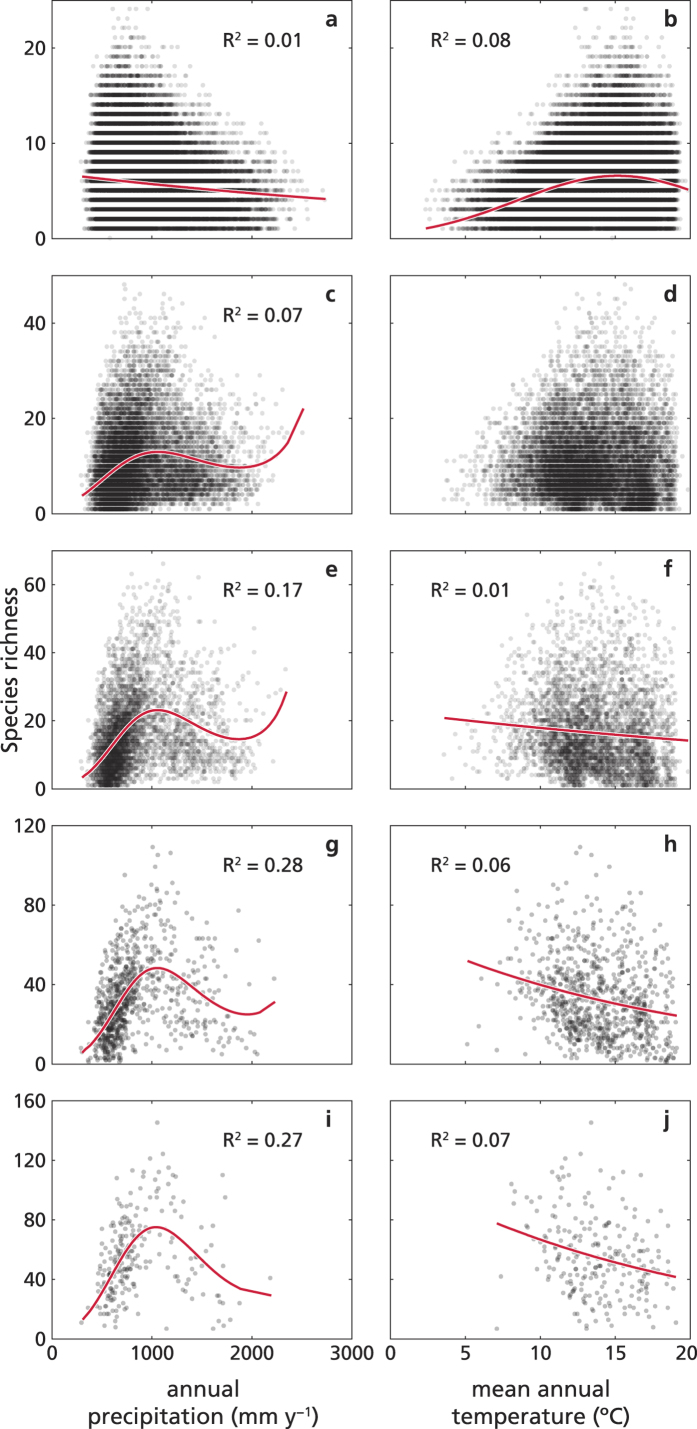
Species richness and fitted models at different scales in the SFI. Species richness (dots) as a function of annual precipitation (left) and mean annual temperature (right), computed from the SFI data at different spatial scales, i.e. (from top to bottom) site scale (**a**,**b**), or equal sized grains of 5 × 5 km (**c**,**d**), 10 × 10 km (**e**,**f**), 25 × 25 km (**g**,**h**), and 50 × 50 km (**i**,**j**). Continuous lines are the selected models (**a**,**f**,**h**,**j**: linear models; **b**,**c**,**e**,**g**,**i**: cubic models; **d**: no model had R^2^ larger than 0.01). See Supplementary Table S2 for details on model selection.

We started exploring the relationships between species richness at site scale and two climatic variables separately (namely, annual precipitation and mean annual temperature). We first analysed the data at site scale (as available from the SFI), and then we processed the site data to generate four new grid-based databases, each consisting of equal sized square cells (5 × 5 km, 10 × 10 km, 25 × 25 km and 50 × 50 km, respectively) for which we computed annual precipitation, mean annual temperature, and the corresponding species richness (i.e. the number of woody plant species present in the sites each cell contains). In mainland Spain, both site and up-scaled species richness values of woody plants varied largely with annual precipitation and mean annual temperature, following highly heteroscedastic patterns with maximums around 700 mm y^−1^ and 13 °C respectively (Fig. 3). At the site scale, the best GLM of species richness (selected as described below) decreased linearly with precipitation, and (quadratically) increased with temperature, but the model explained variance (i.e. the R^2^) was very low. At the upscaled grains of analysis, the R^2^ of species richness were quite low, with temperature explaining a quite small fraction of the variance at all grain sizes, and with precipitation that tended to explain an increasing fraction of the variance when moving from fine to coarse grains (see Fig. 3 and Supplementary Table S2). Models reflected generally consistent trends across grains, i.e. a cubic relationship of richness with precipitation (with an underlying increasing trend), and predominantly a negative linear relationship with temperature. Thus, the models at different grain sizes essentially captured the same climate signals for species richness.

**Figure 4:**
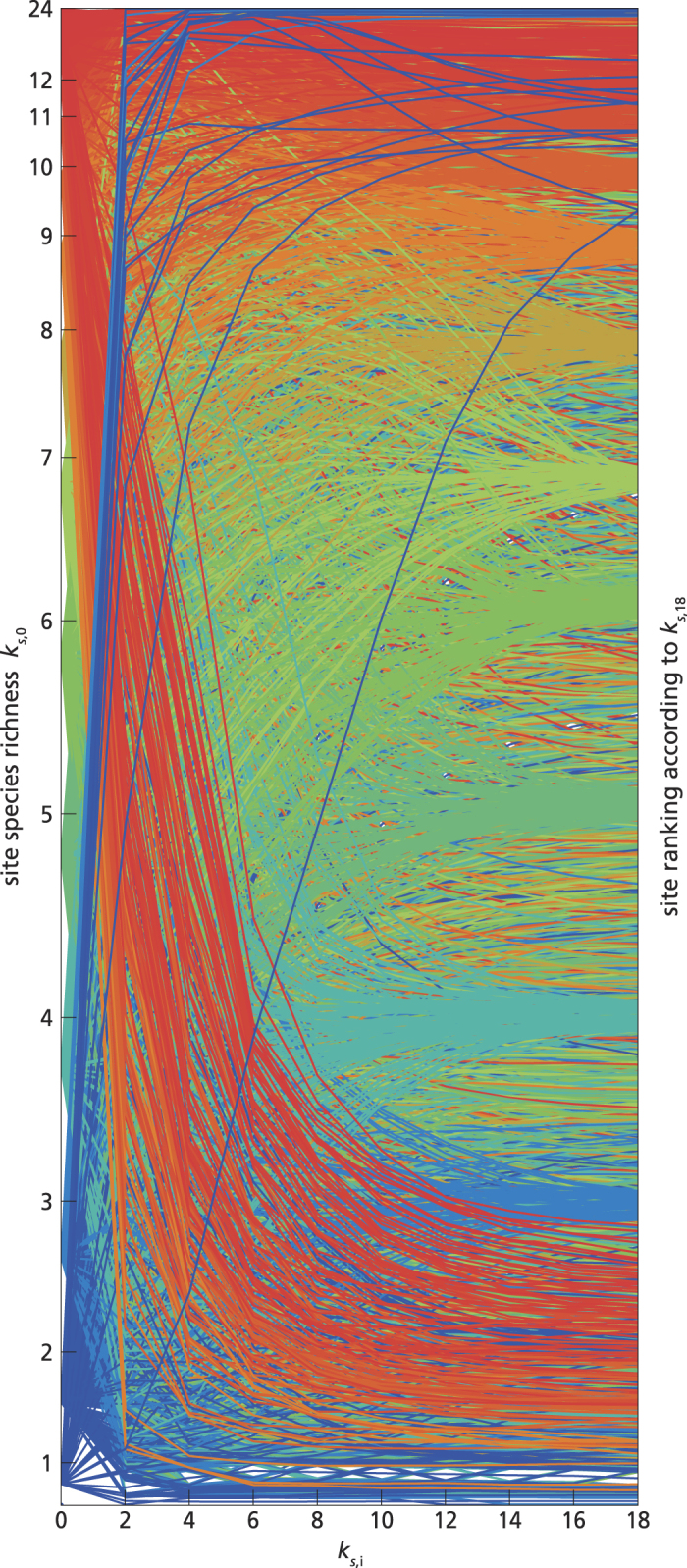
Comparison of site ranking according to successive measures of species richness (even reflections) in the SFI. Sites are ranked according to site species richness *k*_*s,0*_ (to the left of the graph). Each line indicates one site, and the colour is related to the site species richness (red for high and blue for low species richness). Moving towards the right, each site may change ranking according to the reflections of increasing (even) order (x-axis), until *k*_*s,18*_. Overlapping lines (due e.g. to identical species composition of different sites) are reported only once. For clarity of illustration, the lines are represented in the following order, from foreground to background respectively, the “anomalous”, the “typical”, and the remaining sites.

Subsequently, we applied the method of reflections and calculated site and plant species reflections, up to the order *N* = 18 (as in ref. 30). Since the reflections are indices, and their values are not comparable at different order, to relate them we ranked the sites according to each of their even reflections (from *k*_*s,0*_ to *k*_*s,18*_), following ref. 30, and we analysed the patterns of change in ranking. The first aim of this analysis was to observe whether or not the site ranking was changing with increasing order of the reflection. As expected^30^, in the SFI data the rankings tended to converge after a few iterations (e.g. *N* ~ 0–12, see Fig. 4), which also confirmed that the maximum order calculated (*N* = 18) was high enough. The second aim of the rank analyses was to identify which sites were furthest (or closest) to their potential species richness (as explained above in the previous section, and in the Supplementary Note). We could clearly notice (in Fig. 4) that some sites were represented by lines ending up approximately at the same height on the right axes from which they started on the left axis. These sites displayed little change in their ranking (i.e. less than 1% rank change from *k*_*s,0*_ to *k*_*s,18*_), and we considered them as “typical” sites, meaning that the behaviour of their species was similar throughout the network, and their species richness was close to the potential species richness, as expected from their species composition. Other sites instead changed a lot in their ranking position, either from very low species richness (bottom left corner, blue lines) to very high generalized species richness (top right corner), or vice versa from high species richness (red lines) to low generalized values (from top left towards bottom right). We defined as “anomalous” those sites whose rank position changed more than 80%: the method corrected their species richness, identifying it as anomalous with respect to what potentially could be expected given their species composition. To illustrate this, we choose two examples of common tree species that occur largely in the “anomalous” sites (*Fagus sylvatica* and *Pinus halepensis*). *Fagus sylvatica* tends to live with few other woody species. In our dataset, it occurred with less than 5 species in 67% of its sites. No sites including *F. sylvatica* increased their rank, while 42% of the sites where this species co-occurs with more than 10 other species decreased in ranking position. Since *F. sylvatica* behaves as a superior competitor in the study region^38^, it tended to occur in species poor sites, and thus its contribution to calculating generalized species richness was low. Therefore, the sites where *F. sylvatica* was found with many other species decreased in ranking. Another, opposite example is *Pinus halepensis*, which tends to co-occur with many shrub species, mainly in calcareous sites (see e.g. ref. 39). No sites including *P. halepensis* decreased their ranking position, while 80% of the sites where this species occurred on its own, or with one other species, increased their ranking. The method identified these low richness sites as “anomalous”, and “potentially” rich, as indicated by their ranking according to generalized species richness, which was higher than according to observed species richness. In general, we observed that the sites that did not change in ranking (i.e. the “typical” sites) were evenly distributed geographically, while those that decreased in ranking clustered in Northern Spain (a wetter and cooler region with Atlantic macroclimate), and those that increased rank were mostly at the boundary between the semi-arid and arid Mediterranean areas (see the map in Supplementary Figure S4).

**Figure 5:**
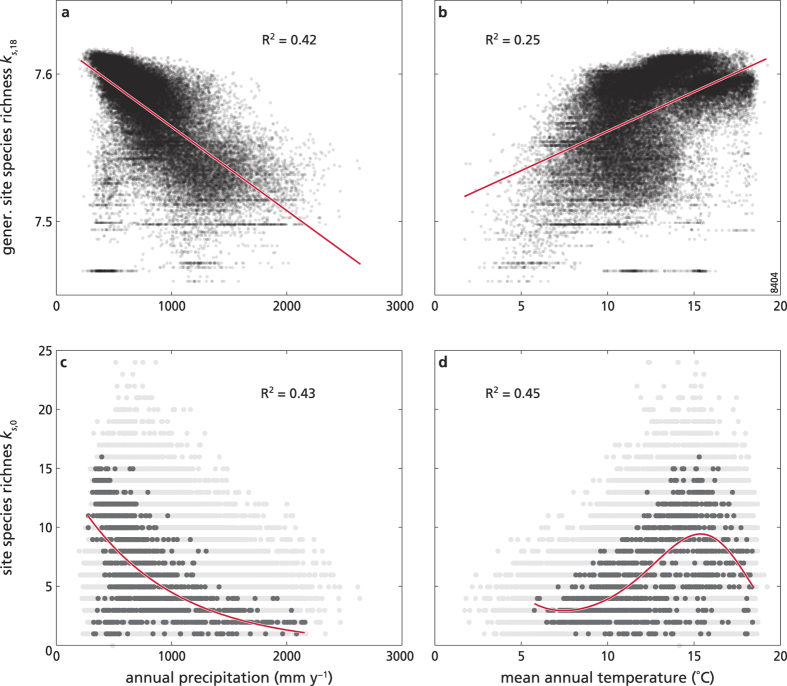
SFI site species richness versus climate. Each dot represents a site reflection as a function of site annual precipitation (mm y^−1^, left panels) and site mean annual temperature (°C, right panels). The top panels (**a**,**b**) show generalized site species richness *k*_*s,18*_, and the selected models (continuous line; linear for both). The bottom panels (**c**,**d**) show species richness of the “typical” sites (black dots), i.e. sites selected according to their small (<1%) change in ranking when going from low (*k*_*s,0*_) to high (*k*_*s,18*_) reflections, with the continuous line representing the selected model (**c**: linear, **d**: cubic). For comparison (in **c** and **d**), the species richness *k*_*s,0*_ of all the other (“non-typical”) sites is also reported (grey dots; same as in Fig. 1a,b). See Supplementary Table S3 for details on model selection.

Finally, we explored the relationship between generalized site species richness (*k*_*s,18*_) and the climate site variables. Linear models (selected following the procedure described in the last subsection of this paper) were the best fit for both site annual precipitation and mean annual temperature (see Supplementary Table S3). Generalised site species richness decreased strongly with increasing site mean annual precipitation (R^2^ = 0.42, Fig. 5a), while it increased with site mean annual temperature, although with a less good fit (R^2^ = 0.25, see Fig. 5b). In other words, generalized site species richness (*k*_*s,18*_) was higher in arid (dry and warm) sites (i.e. under characteristic Mediterranean macroclimate conditions). We then focused only on the “typical” sites (i.e. those with less than 1% change in ranking from the lowest to the highest reflection), and we analysed the relationship of their site species richness (*k*_*s,0*_) with the climate variables. We expected that woody species richness of these sites would follow the same pattern of the generalized species richness of all the sites, because the “typical” sites should not change substantially in their behaviour going from low to high reflection order. We found that the species richness (*k*_*s,0*_) of the “typical” sites displayed the same general trend as the generalized species richness of all the sites. For the selected sites, species richness decreased as a function of annual precipitation (the log-linear model was the best fit, with R^2^ = 0.43, see Fig. 5c), and increased with mean annual temperature for most of the range (the best model being a cubic relationship, with R^2^ = 0.45, see Fig. 5d). We obtained similar results if we defined the “typical” sites using different tolerance intervals (e.g. within 5% or 10% change in ranking, not shown). For further details on model selection, see Supplementary Table S3.

**Figure 6:**
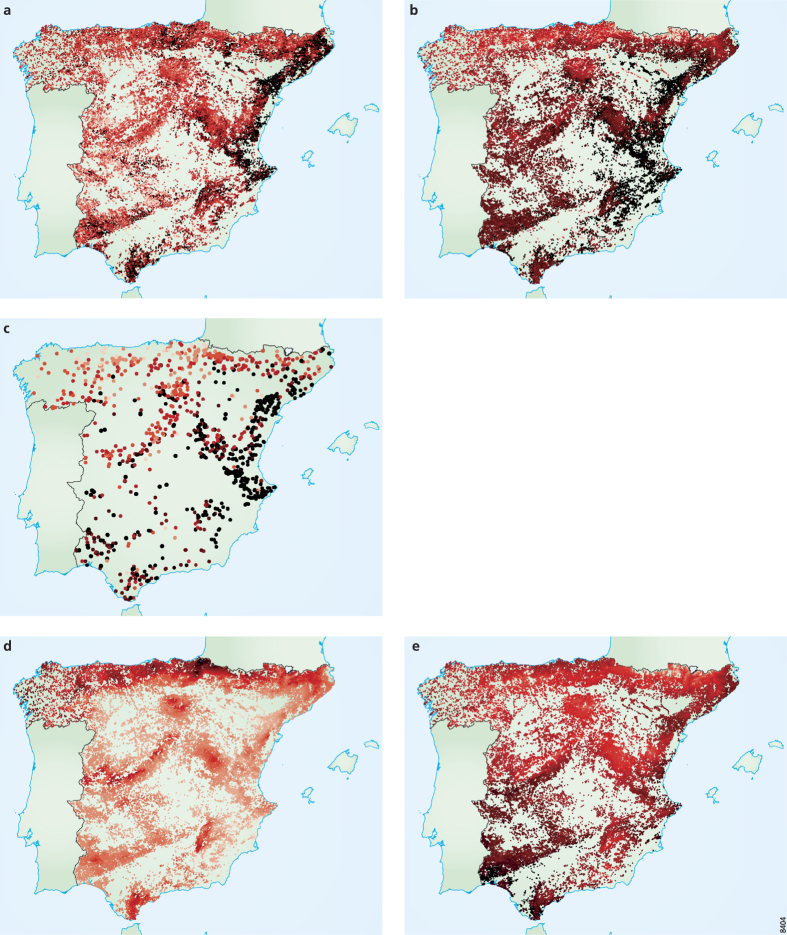
Maps of Spanish plant species richness and climate. In each map, darker colours represent higher values of the represented variables, respectively: site species richness, *k*_*s,0*_ (**a**); generalized site species richness, *k*_*s,18*_ (**b**); species richness (*k*_*s,0*_) of the “typical” sites (**c**); site annual precipitation (**d**); site mean annual temperature (**e**). Generalized species richness and species richness of the “typical” sites (panel **b** and **c**, respectively) display similarity with the patterns of annual precipitation and mean annual temperature (panel **d** and **e**, respectively), while species richness (panel **a**) displays less similarities when inspected by eye (see e.g. the region in the centre-west of Spain, displaying noisy species richness data including many minimal values, while temperature and precipitation display quite uniform high and low values, respectively). Note that in panel **c** dots are larger than in the other panels for illustrative purposes only (since the number of selected “typical” sites is less than 10% of the total). The maps where produced with Adobe Illustrator with the MaPublisher plugin.

The noise removal was also apparent when comparing the map of site species richness (Fig. 6a), with those of generalized species richness (*k*_*s,18*_, Fig. 6b) and of species richness (*k*_*s,0*_) of the “typical” sites (Fig. 6c). While the two latter maps show patterns analogous to those of precipitation and temperature (Fig. 6d,e), the site species richness map (Fig. 6a) follows the climate patterns less clearly when inspected by eye. For example, in the centre-west region of Spain, sites display a wide range of species richness, with many low values (light colours, Fig. 6a), while the high temperature (dark colours, Fig. 6d) and low precipitation (light colours, Fig. 6e) are quite uniform throughout the area.

## Discussion

In this work we introduced the method of reflections, a new network technique that successfully isolated the site species richness signal, incorporating at the local level information about the distribution of species from the whole dataset. The method introduced the generalised species richness: a new index that is related to potential species richness. We applied this method to study how woody plant species patterns in mainland Spain relate to precipitation and temperature gradients, finding out that large part of the variation of site (potential) species richness could be explained with annual precipitation (and, to a lesser extent, mean annual temperature). At the local site scale, the method of reflections showed that woody communities in drier and warmer areas tended to have higher richness of woody plant species, and, remarkably, we observed the same signal in the generalized species richness of all the sites, and in the (non-processed) species richness of a sub-set of sites that we identified as “typical”. Interestingly, these trends largely coincided with those found by Terradas *et al.*^31^ in their analysis of woody species richness variation across canonical, well-preserved woodland communities in NE Spain (for more comparable results, see also ref. 40). In contrast, when analysing the data using the classical geographical upscaling, the trends revealed were different (i.e., a third-order polynomial relationship of richness with annual precipitation, with an overall increasing trend, and a predominantly negative relationship with mean annual temperature), similarly to what Vetaas & Ferrer-Castán^32^ reported in their grid-based analysis of woody species richness patterns in the Iberian Peninsula.

The new index we introduced here, the “generalised species richness”, was closely related to site potential species richness. The index combined information in a novel manner, taking species distribution and the inter-species structure into account. The new definition was based on within-network similarities (i.e. community assembly patterns), including information from sites that had similar species composition, irrespective of their geographical locations. The method actively reduced noise and isolated the signal, including information from the whole dataset at the local (site) scale, and introducing a sort-of weighted averaging technique, in a way reminiscent of e.g. Principal Component Analyses, but with the advantage of not using information from the explanatory variables. Comparing all the communities with similar taxonomic compositions, the method calculated the generalized species richness of a certain site as expected given its current species composition. The method recognized the sites with uncommon richness as outliers with respect to their species composition, reducing the noise they create, and allowing small-scale data to gain greater generality. In this process, the method recognized whether each species in a certain site lived with an anomalous number of other species. However, it did not recognize whether or not these species are always the same throughout the dataset. It is worth remarking that the sites (and their communities) were identified as “anomalous” only because they do not represent the most typical situations, without any implication about their ecological relevance.

As in the case of the product exports of countries^30^, the method of reflections proved to be able to determine key relationships underlying the network structure also for the woody plant distribution in mainland Spain. Here, the method showed the climate fingerprint in the species distribution very clearly, and confirmed our expectation (derived from the findings of Terradas and coauthors^31^, see above) that community level woody species richness tends (potentially) to be higher in the more arid Mediterranean communities. While site species richness in the dataset exhibited only negligible relationships with precipitation and temperature, these climate variables could fit much better the generalized species richness (with very good fit performance, especially for precipitation).

The method identified some sites as anomalous in woody Spanish forests, possibly due, for example, to the existence of extraordinary environmental conditions (e.g. atypical soil characteristics), or to their history of anthropogenic disturbances, which could have lead the local community to be species richer or poorer than potentially expected. Checking the method performance with a few specific examples, we observed that the sites displaying more species than expected often corresponded to sites with *F. sylvatica* that (for reason such as soil heterogeneity, ungulate grazing, etc.) appear richer than common *F. sylvatica* forests, typically poor in woody plants because of the superior competitive abilities of this species^38^. On the contrary, the “anomalous” sites corresponded at large to Mediterranean forests, most of them with *P. halepensis*, at the boundary between the semi-arid and arid areas of Southeast Iberia, or influenced by the Ebro depression. In these sites, the common trend of *P. halepensis* dominated forests, i.e. exhibiting a rich cohort of shrub species (e.g. ref. 39), was not present, suggesting that unaccounted environmental or management factors (e.g. fire, grazing or planted origin) had prevented these communities to reach their potential species composition. This was not connected only to these two species and to their communities, which we chose as illustrative examples. In general, there was a tendency for the “anomalous” sites occurring in the Atlantic region of northern Spain to have more species than expected, while the “anomalous” sites of the Mediterranean region exhibited fewer species than expected. In a way, we can say that “noise” and “disturbances” act in the direction of levelling off the species richness differences along the gradient in Spanish forests.

These examples, together with the application to synthetic datasets, illustrate that the method identifies the sites that deviate from most common or “potential” assemblages. These deviations could be due to different underlying processes, such as historical legacies (e.g. land use history or forest management), environmental heterogeneity (e.g. slope aspect, edaphic conditions), trophic interactions (e.g. herbivory, pathogens) or community dynamics (e.g. successional stage). Thus, the method could be used to identify hotspots of species richness (identifying those sites that are richer than potentially expected), or areas where management could be particularly effective in enhancing species richness (i.e. sites where species richness is currently lower than expected). This information could be usefully applied in community or restoration ecology (see also ref. 41), as it allows to infer an indication of potential plant richness for a given site not on the basis of climatic preferences (as in classical gradient analyses), but rather of environmental and/or endogenous mechanisms driving community organization. This kind of application could be previously validated using datasets with different historical records.

The method of reflections is an alternative to classical methods to look at species richness patterns. For example, geographical upscaling aggregates local data at coarser grains, according to their geographical proximity. This is an effective way to smooth out local environmental heterogeneities, and it renders species richness data comparable to larger scale climate variables. However, many studies have found not only that the predictive power of environmental predictors often changes with grain size (e.g. ref. 42,43), but also that particular richness-environment relationships may even show different sign depending on the size of the analysis units used (e.g. refs. 13,33). As pointed out by Rahbek & Graves^44^, the use of coarse grains introduce an averaging effect that obscures the fine structure of species richness gradients and localized richness peaks, thus decreasing our possibilities to discriminate the causal agents underlying richness patterns.

Although aggregating local data at coarse grain is in a way similar to what the method of reflections does (i.e. removing noise), our method identifies the local species richness signal, and brings out information about potential species richness, integrating information from the species community composition in the whole dataset, instead of using geographical proximity and calculating species richness at coarser grain. For this reason, in our case study, the method of reflections could identify strong relationships of (generalized) species richness with precipitation and temperature (negative and positive, respectively), indicating that local woody communities in dryer and warmer areas across Spain, corresponding to the Mediterranean ecosystems, can potentially have higher woody plant species richness.

The method of reflections showed a very high performance in catching the main factors of variability underlying the dataset analysed, and allowing an effective integration of ecological variability to obtain indications of potential species richness. Since presence/absence datasets are very common in ecological data collection, the method could be applied to analyse other geographical areas, other trophic levels or species groups at various scales, possibly leading to very interesting ecological insights on the environmental drivers of species distributions and richness, due to a method that elaborates species richness information on the basis of co-occurrence patterns and community structure rather than on geographical or environmental proximity.

## Methods

In the following two subsections, we describe respectively the datasets we used for the analyses of the Spanish woody species, and the use of GLM and the model selection procedures. All the analyses in this work were performed using MATLAB R2013b (The MathWorks, Inc.).

### The Spanish study area and the datasets

In this paper we analysed data from an extensive dataset describing woody species of continental Spain. This region (492,173 km^2^) comprises large altitudinal (max. 3500 m.a.s.l.) and climatic gradients (from Atlantic to Mediterranean climate), and consequently displays a large diversity of habitats and species. We used the third Spanish Forest Inventory (SFI), which was surveyed between 1997 and 2007, distributing a 1-km^2^ cell grid over Spain^45^. The data used to compute the presence-absence in each site, and thus to build the matrix for the network analysis, were obtained differently for trees and shrubs from the SFI dataset^45^. For trees, the set-up at each site involved four concentric circular sub-plots of 5, 10, 15 and 25 m radius each, which measured individuals with d.b.h. (diameter at breast height) larger than 7.5 cm, 12.5 cm, 22.5 cm and 42.5 cm, respectively in each of the subplot. For shrubs, species presence-absence was recorded at the 10 m radius subplot.

We selected a total of 45,620 sites of the SFI according to the following criteria: (i) being located over mainland Spain; (ii) having at least one adult tree; (iii) showing no evidence of thinning or harvesting; and (iv) not being identified as planted. We obtained the planted character from ref. 46, where the map of Spanish Provenance Regions of Forest Species was joined spatially with the SFI sites, using a definition of planted forests as not originated by natural regeneration from local or nearby native sources^47^,^48^. We included exotic species, because they might influence the distribution of native plants, but excluded all species present in less than 10 sites, obtaining a total of 211 species for the analysis (see Supplementary Table S4). Note that, because we focused on forests with canopy dominant trees, woody species that tend to occur only in open habitats but not in early successional forests might be under-represented.

We considered mean annual temperature and annual precipitation to represent the major climatic conditions of each site in mainland Spain, with climatic variables obtained at 1 × 1 km scale (series 1951–1999, ref. 49). We selected these two climatic variables as representative of the climatic conditions in each site following previous studies^38^,^46^, which showed (using Principal Component Analyses) that these two variables contain the largest environmental variation among an initial set of potentially correlated topographic and climatic variables of the Iberian Peninsula. These two variables are weakly correlated (*r*^2^ = 0.12), thus minimizing multicollinearity.

### GLMs and model selection

To explore the relationships between variables in this work (e.g. species richness and climatic variables), we took the following steps (see also e.g. ref. 32). We used polynomial Generalized Linear Models (GLMs), with: i- log link and a Poisson error distribution for count data (such as species richness); and ii- identity link and a normal error distribution for continuous data (such as generalised species richness). We tested functional forms including from linear up to cubic terms of the explanatory variables. For each model, we calculated the pseudo-R^2^ (as in ref. 50) (which for brevity we call R^2^ in this paper), and the corrected Akaike Information Criterion (AICc^51^). For each grain size and each climatic variable, we selected the best model with the following criteria, which tried to maximize parsimony and model fit at the same time, keeping the model complexity as low as possible^52^. Starting from the linear model, we would select a model with higher order (quadratic or cubic) only if: i- R^2^ increased at least of 0.01 when increasing order, and ii- the AICc index of the more complex model was at least two unit lower^53^. We chose not to select any model if none of them had R^2^ larger than 0.01.

## Additional Information

**How to cite this article**: Baudena, M. *et al.* Revealing patterns of local species richness along environmental gradients with a novel network tool. *Sci. Rep.* **5**, 11561; doi: 10.1038/srep11561 (2015).

## Supplementary Material

Supplementary Information

## Figures and Tables

**Table 1:** Explanation of the different variables (“reflections”) obtained with the method of reflections, including node type used, reflection symbol, parity, order and name

**Node type**	**Reflection symbol**	**Parity**	**Symbol including order**	**Name**	**Explanation**
Sites	*k*_*s*_	*Even*	*k*_*s,18*_	Generalized site species richness	How species-rich is the site? *Species living on average in richer sites count more*
		*Odd*	*k*_*s,17*_	Generalized frequency of occurrence of the species living in the site	How widespread are the species present at the site? *Species living (on average) in association with other widespread species count more*
plant species	*k*_*p*_	*Even*	*k*_*p,18*_	Generalized species frequency of occurrence	How widespread is the species? *Sites including other widespread species count more*
		*Odd*	*k*_*p,17*_	Generalized species richness of the sites where the species live	How species-rich are the sites where the species lives? *Sites with species living in richer sites count more*
