# Supplementary material for: Revealing patterns of local species richness along environmental gradients with a novel network tool
Source: Sci Rep. 2015 Jun 25;5:11561. doi: 10.1038/srep11561 (PMC4479799; doi:10.1038/srep11561)
Supplement: Supplementary Information [file srep11561-s1.doc]

**Supplementary Information of:**

*Revealing patterns of local species richness along environmental gradients with a novel network tool*

Mara Baudena, Angel Sánchez, Co-Pierre Georg, Paloma Ruiz-Benito, Miguel Á. Rodríguez, Miguel A. Zavala, Max Rietkerk

Here we present the following:

- Supplementary Note. An illustrative application to synthetic datasets p. 1-4

- Other Supplementary Figures and Tables p. 5-12

# Supplementary Note

*An illustrative application to synthetic datasets*

We show here the supplementary information relative to the section “An illustrative application to synthetic datasets” in the main text.


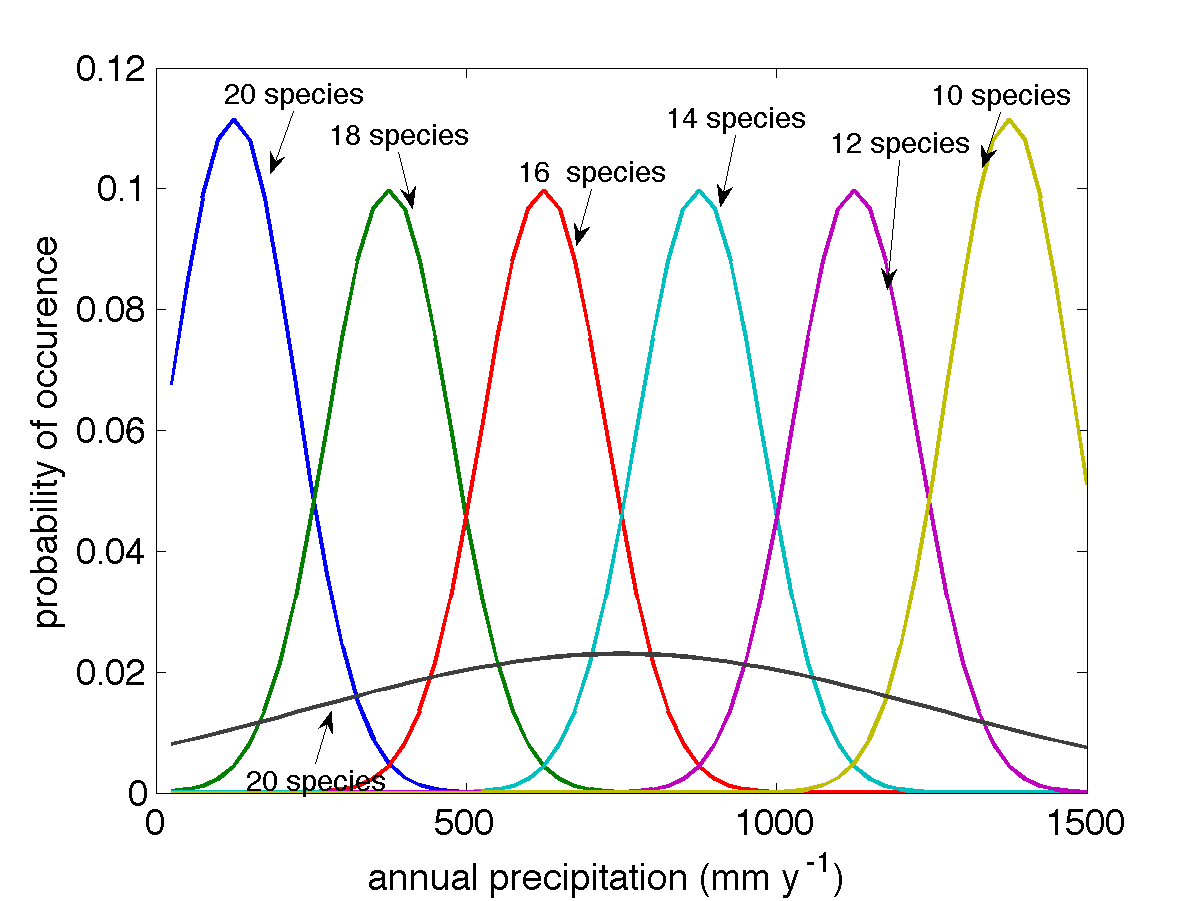


Fig. S1 Dataset D1, species probability of occurrence. Species occur randomly in the sites, with probability of occurrence that varies with the site annual precipitation as shown here. The distributions are normal, truncated between 0 and 1500 mm y^-1^. 20 species have the most leftward distribution, 10 the most rightward, linearly decreasing in between. 20 species are generalists (wide distribution, black line at the bottom of the graph).

*Species “sociability” and the generalised species richness*

We show here with the dataset D1 (see description in the main text) that the method of reflections uses information about the “sociability” of the species (i.e. how likely they are to live with other species) to calculate the generalised species richness, which, as we have seen in the main text, is related to the potential species richness of a site. In this example, we can evaluate the theoretical “sociability” of each species, since we know the theoretical probability of occurrence of all the species. For the species *i*, in each site *j* where its probability of occurrence is finite (prob*_i_*(*j*)>10^-6^), we sum up the probabilities of all the other species *l*:

$q_{i}(j)=\sum_{l\neq i} \mathrm{prob}_{l}(j)$,

and then we calculate the average of this value over all the sites *j*, obtaining for each species

$S_{i}=\mathrm{mean}(q_{i}(j))$.

We call here *S_i_* “sociability” of the species *i*, because it is proportional to the likelihood of that species to live with others. If we average the species “sociability” *S_i_* over all the species in a site, we therefore obtain a measure of the potential species richness of the site, as given by the tendency of the species present in the site to be “social”. The average species “sociability” per site turns out to grow linearly with the generalised species richness *k_s,18_*, with a very high fraction of variance explained (Fig. S2, R^2^=0.98). This confirms our explanation of the method derived via the mathematical formulas (Eqs 3-4 and Table 1 in the main text), showing that the generalised species richness (*k_s,18_*) is a measure of the potential richness of the site, as calculated keeping into account the “sociability” of the species present in that site. In other words, the generalised species richness uses information from sites with similar species composition, since the species “sociability” is calculated from the pattern of occurrence of the species in all the sites.


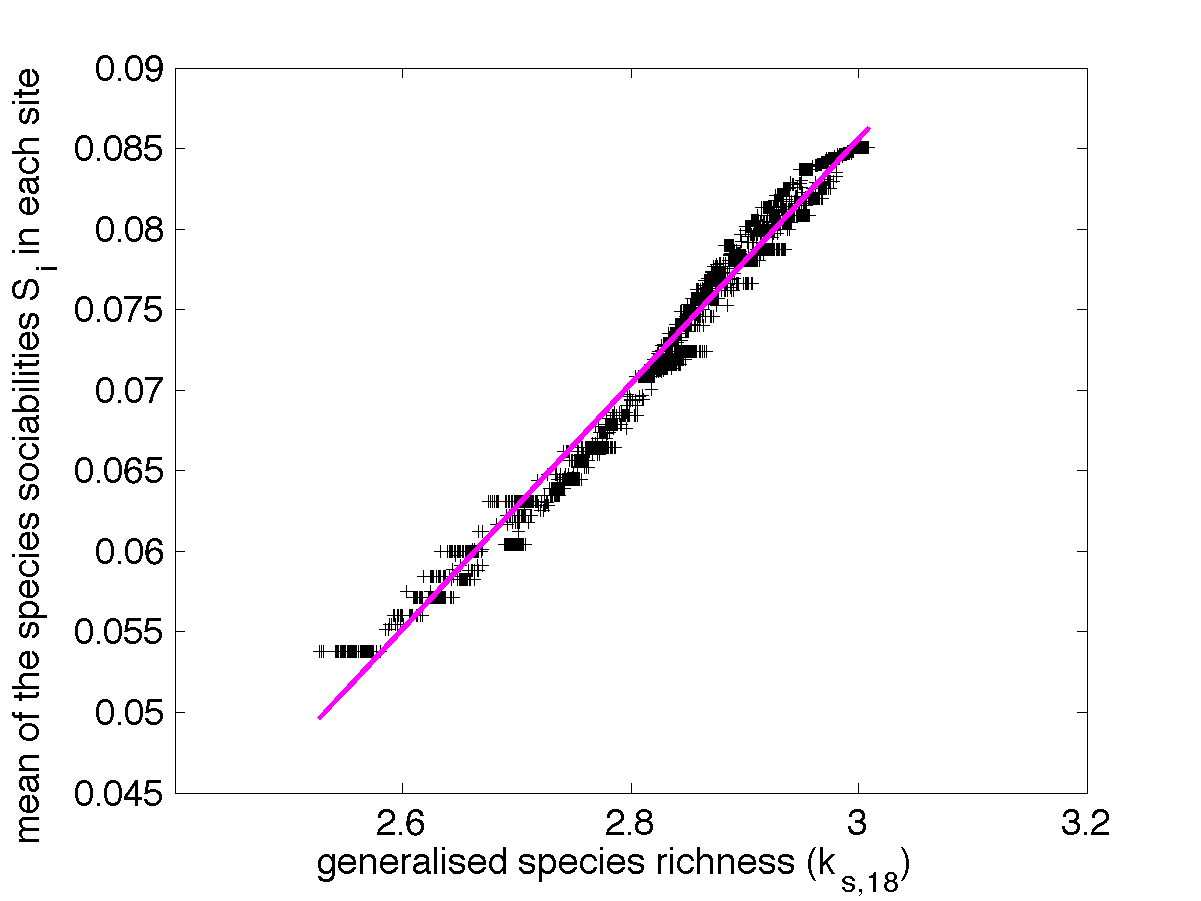


Fig. S2 Mean of the “sociability” *S_i_* of the species in each site, as a function of *k_s_*_,18_ (dataset D1). Each cross represents a site. The continuous line is the linear model prediction (R^2^=0.98, see Table S1)

*Rank changes represent distance from potential richness*

Using the same dataset (D1) we also show here that the site changes in ranking from generalised species richness *k_s_*_,18_ to species richness *k_s_*_,0_ represent how far (or close) sites are from their potential richness.

Since for each species we knew the corresponding theoretical probability of occurrence in each site (given the site annual precipitation), we used these probabilities to quantify the distance of each site from the potential richness. In detail, we quantified this distance using the sum of the probability of occurrence of all the absent species, normalised by the sum of the probability of occurrence of all the species in the site (i.e., the sum of the species probabilities of both absent and present species). Thus, we had a proportional measure of how far the actual species composition of a site was from the potential, as we divided the (sum of) probability of occurrence of the species not observed by the (sum of) probability of occurrence of all the species in the site, i.e. we normalised for sites that are potentially species rich or poor. When plotting this site distance from potential species richness as a function of the site rank change for the dataset D1, we found that their best fit is an increasing linear trend, with fairly high fraction of the variance explained (R^2^=0.54, Fig. S3).

We can thus state that sites with very large, positive, rank change (i.e. sites that increase in ranking from species richness to generalised species richness) have much larger potential species richness than observed. Vice versa, sites with very large and negative rank change (i.e. sites that decrease in ranking from species richness to generalised species richness) are sites whose species richness is close to potential, i.e. only few extra species could occur in the site but are not actually observed.


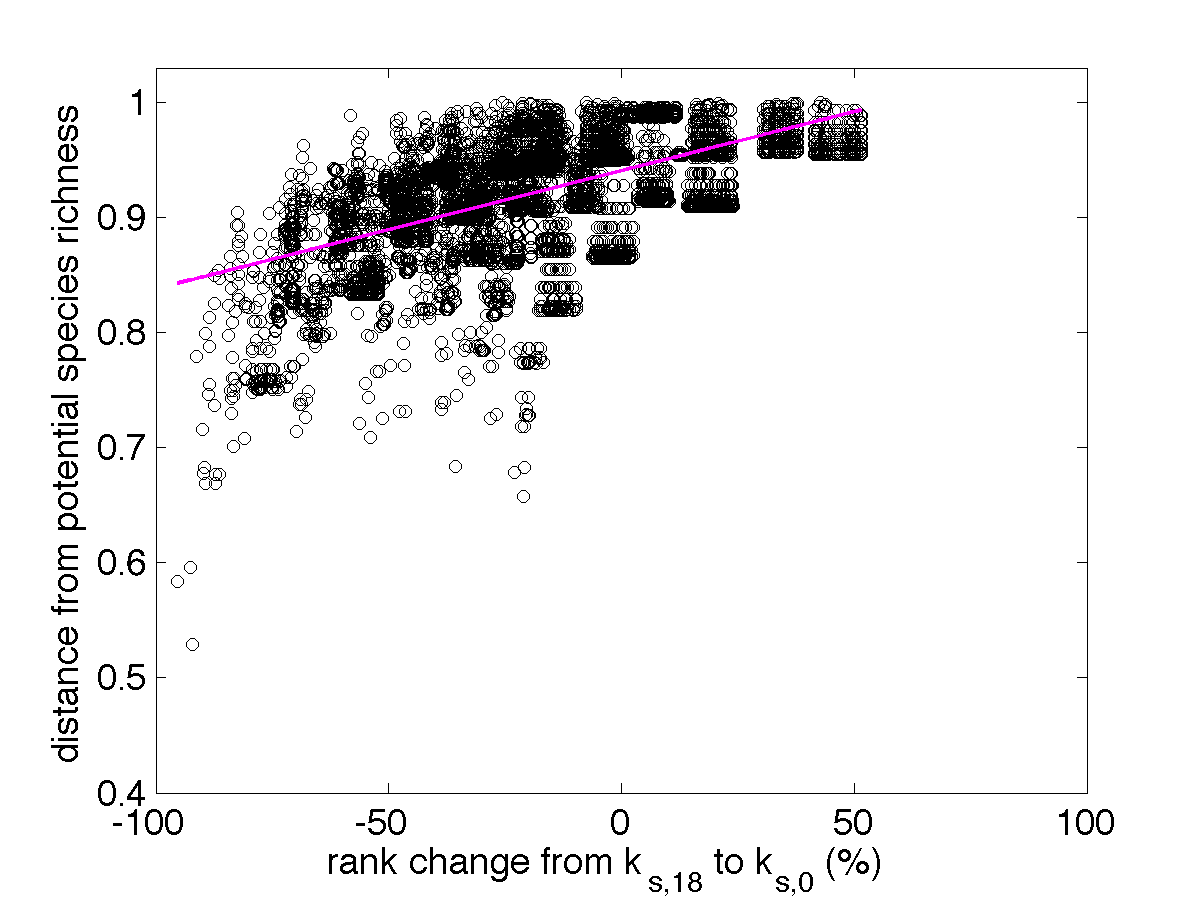


Fig. S3 The distance of the sites from potential richness (calculated as the sum of the probabilities of occurrence of the absent species, normalised to the sum of the probabilities of occurrence of all the species in the site) plotted as a function of the site change in ranking from species richness *k_s_*_,0_ to generalised species richness *k_s_*_,18_ for dataset D1 (best fit, linear trend, R^2^=0.54, see Table S1).

Table S1 Results of the different GLM alternative model for the dataset D1, corresponding to the trend lines in the Fig. 2 (main text), and Supplementary Fig. S2-S3. (1) Species richness versus annual precipitation (mm y^-1^), Fig. 2 a; (2) generalised species richness versus annual precipitation (mm y^-1^), Fig. 2 c; (3) mean “sociability” of the species in each site versus generalised species richness, Fig S2; (4) distance of a site species richness from potential versus rank change, Fig S3. For each of the explanatory (x) and dependent (Y) variables, three models are tested: linear (Y=a+bx), quadratic (Y=a+bx+cx^2^), and cubic (Y=a+bx+cx^2^+dx^3^). For continuous data (e.g. generalised species richness), we assumed Normal distribution of errors, while for count data (i.e. species richness), we assumed Poisson distribution of errors. Mc Fadden pseudo-R^2^, and corrected Akaike Information Criterion (AICc) are reported for each case. The selected models (represented in the figures) are marked in bold characters for each figure panel. See Method section in the main text for a detailed description of the GLM and the model selection procedures. The units of the parameters used in the fits are reported as a function of the units of the explanatory x-variables.

| Dependent variable and figure | GLM error distr. | Model parameters | | | | R^2^ | AICc |
| --- | --- | --- | --- | --- | --- | --- | --- |
|  |  | a  [-] | b  [x^-1^] | c  [x^-2^] | d  [x^-3^] |  |  |
| (1) Species richness  (Fig. 2 a) | Poisson | 0.93 | -4.5E-04 | - | - | 0.06 | 11237 |
|  |  | **0.72** | **3.9E-04** | **-5.8E-07** | **-** | **0.07** | **11077** |
|  |  | 0.73 | 3.0E-04 | -4.2E-07 | -7.2E-11 | 0.07 | 11079 |
| (2) General. species richness  (Fig. 2 c) | Normal | **3.0** | **-2.7E-04** | **-** | **-** | **0.84** | **26** |
|  |  | 3.0 | -1.1E-04 | -1.1E-07 | - | 0.86 | 26 |
|  |  | 3.0 | -1.2E-04 | -8.9E-08 | -8.8E-12 | 0.86 | 28 |
| (3) Mean sp. “sociability” in sites (Fig. S2) | Normal | **-0.2** | **8.1E-02** | **-** | **-** | **0.98** | **6** |
|  |  | 0.08 | -8.5E-02 | 3.0E-02 | - | 0.99 | 8 |
|  |  | 11.0 | -11.8 | 4.2 | -0.50 | 0.99 | 10 |
| (4) Dist. from potential richness  (Fig. S3) | Normal | **0.94** | **1.0E-03** | **-** | **-** | **0.54** | **20** |
|  |  | 0.95 | 7.1E-04 | -1.1E-05 | - | 0.58 | 21 |
|  |  | 0.94 | 5.6E-04 | -6.4E-06 | 9.3E-08 | 0.58 | 21 |

**Other supplementary figures and tables**

*(In the order they are mentioned in the main text)*

**Table S2**

**Results of the Generalized Linear Model (assuming Poisson distribution of errors) between species richness (S) at five different grain sizes (sampling plot and square cells of 5x5, 10x10, 25x25 and 50x50 km) and the associated climate variables, namely annual precipitation (Prec, mm y^-1^) and mean annual temperature (Temp, ^o^C). For each explanatory variable (x), three models are tested: linear (S=a+bx), quadratic (S=a+bx+cx^2^), and cubic (S=a+bx+cx^2^+dx^3^). Mc Fadden pseudo-R^2^ and corrected Akaike Information Criterion (AICc) are reported for each case. The selected models (represented in Fig. 3) are marked in bold characters for each climate variable at each grain. The units of the parameters used in the fits are reported as a function of the units of the explanatory x-variables.**

| Grain | Climate variable | Model parameters | | | | R^2^ | AICc |
| --- | --- | --- | --- | --- | --- | --- | --- |
|  |  | a  [-] | b  [x^-1^] | c  [x^-2^] | d  [x^-3^] |  |  |
| Plot | Prec | **1.9** | **-1.8E-04** | **-** | **-** | **0.01** | **78426** |
|  |  | 1.7 | 2.6E-04 | -2.3E-07 | - | 0.02 | 78137 |
|  |  | 1.6 | 7.3E-04 | -7.1E-07 | 1.5E-10 | 0.02 | 78106 |
|  | Temp | 1.2 | 4.1E-02 | - | - | 0.05 | 75810 |
|  |  | **-0.5** | **3.2E-01** | **-1.1E-02** | **-** | **0.08** | **73300** |
|  |  | 1.2 | -1.2E-01 | 2.7E-02 | -1.1E-03 | 0.08 | 72984 |
| 5x5 km | Prec | 2.2 | 2.6E-04 | - | - | 0.02 | 52846 |
|  |  | 1.4 | 2.2E-03 | -9.8E-07 | - | 0.06 | 50693 |
|  |  | **0.4** | **5.5E-03** | **-4.4E-06** | **1.1E-09** | **0.07** | **50020** |
|  | Temp | 2.4 | -6.5E-04 | - | - | 0.00 | 53879 |
|  |  | 1.7 | 1.0E-01 | -4.1E-03 | - | 0.00 | 53724 |
|  |  | 2.0 | 4.4E-02 | 9.9E-04 | -1.4E-04 | 0.00 | 53723 |
| 10x10 km | Prec | 2.5 | 4.1E-04 | - | - | 0.05 | 29534 |
|  |  | 1.2 | 3.5E-03 | -1.6E-06 | - | 0.15 | 26455 |
|  |  | **-0.2** | **8.7E-03** | **-7.0E-06** | **1.7E-09** | **0.17** | **25610** |
|  | Temp | **3.1** | **-2.4E-02** | **-** | **-** | **0.01** | **30747** |
|  |  | 2.8 | 2.3E-02 | -1.9E-03 | - | 0.01 | 30731 |
|  |  | 2.4 | 1.5E-01 | -1.3E-02 | 3.0E-04 | 0.01 | 30726 |
| 25x25 km | Prec | 3.2 | 4.1E-04 | - | - | 0.06 | 9136 |
|  |  | 1.6 | 4.2E-03 | -2.0E-06 | - | 0.24 | 7360 |
|  |  | **0.2** | **9.5E-03** | **-7.6E-06** | **1.8E-09** | **0.28** | **7031** |
|  | Temp | **4.2** | **-5.4E-02** | **-** | **-** | **0.06** | **9187** |
|  |  | 3.9 | -1.1E-02 | -1.7E-03 | - | 0.06 | 9183 |
|  |  | 3.6 | 7.9E-02 | -9.4E-03 | 2.1E-04 | 0.06 | 9184 |
| 50x50 km | Prec | 3.9 | 1.9E-04 | - | - | 0.02 | 2927 |
|  |  | 2.3 | 3.9E-03 | -1.9E-06 | - | 0.25 | 2242 |
|  |  | **1.2** | **7.8E-03** | **-6.1E-06** | **1.4E-09** | **0.27** | **2171** |
|  | Temp | **4.7** | **-5.2E-02** | - | - | **0.07** | **2762** |
|  |  | 4.7 | -6.0E-02 | 2.9E-04 | - | 0.07 | 2764 |
|  |  | 2.9 | 4.0E-01 | -3.8E-02 | 1.0E-03 | 0.08 | 2758 |

**Figure S4**

Map of the location of the most and least “anomalous” sites.


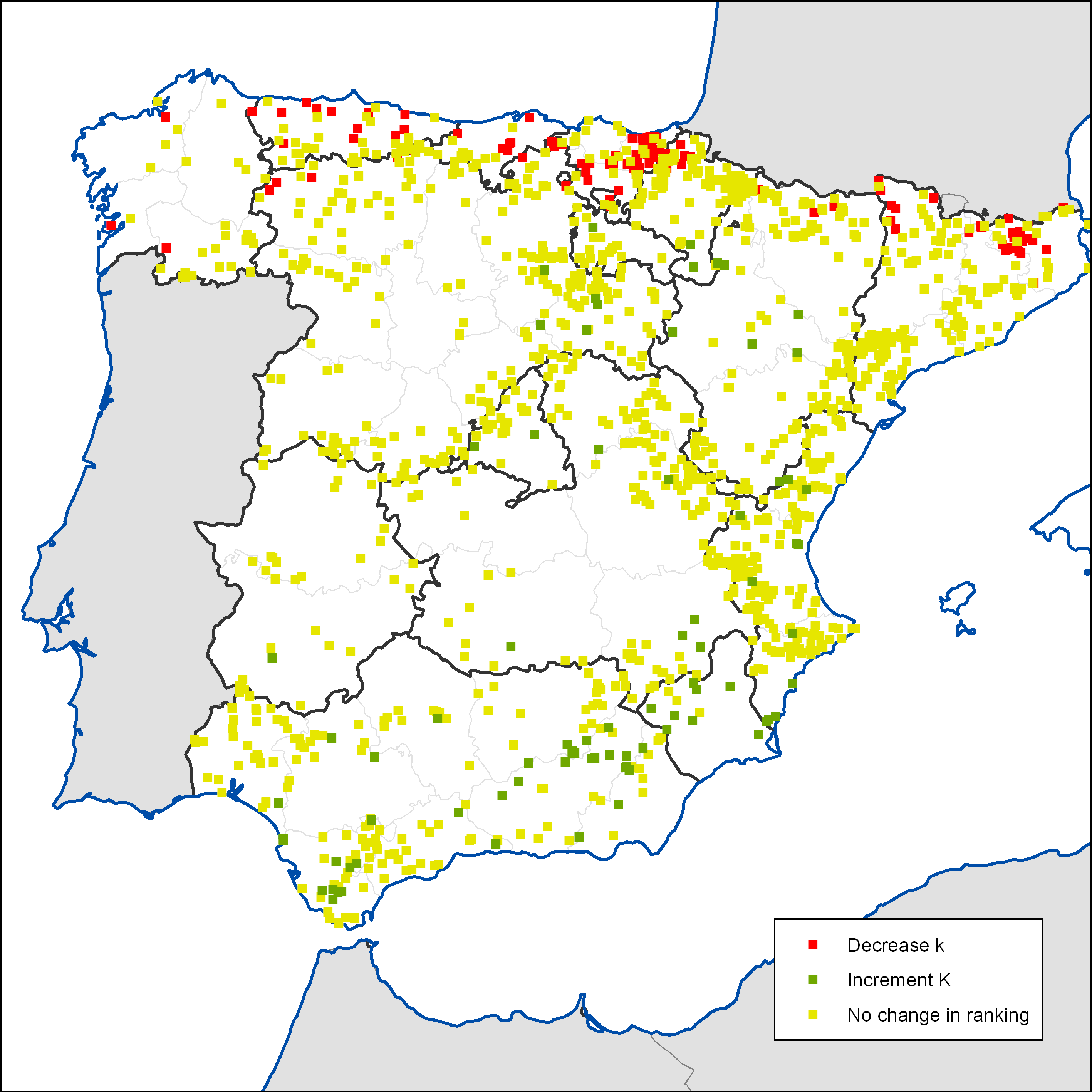


Fig. S4 Map of the location of the least “anomalous” sites (within 1% change in ranking when going from low to high reflection order, yellow squares), and the most “anomalous” (more than 80% change in ranking). The latter are subdivided into sites that increase in ranking (green squares), and sites that decrease in ranking (red squares). The map was produced with ARCVIEW GIS 9.2 (ESRI Inc., Redlands, CA,USA, 2000).

**Table S3**

**Results of the Generalized Linear Model between (1) generalized species richness of all the sites, (2) species richness of the selected “not-anomalous” sites and the associated climate variables, annual precipitation (Prec, mm y^-1^) and mean annual temperature (Temp, ^o^C). For each of the explanatory (x) and dependent (Y) variables, three models are tested: linear (Y=a+bx), quadratic (Y=a+bx+cx^2^), and cubic (Y=a+bx+cx^2^+dx^3^). For continuous data (i.e. generalised species richness, (1)), we assumed Normal distribution of errors, while for count data (i.e. species richness, (2)), we assumed Poisson distribution of errors. Mc Fadden pseudo-R^2^, and corrected Akaike Information Criterion (AICc) are reported for each case. The selected models (represented in Fig. 5) are marked in bold characters for each climate variable and each reflection. The units of the parameters used in the fits are reported as a function of the units of the explanatory x-variables.**

| Reflection | Climate variable | Model parameters [units] | | | | R^2^ | AICc |
| --- | --- | --- | --- | --- | --- | --- | --- |
|  |  | a  [-] | b  [x^-1^] | c  [x^-2^] | d  [x^-3^] |  |  |
| (1) generalized species richness of all sites | Prec | **7.6** | **-5.7E-05** | **-** | **-** | **0.42** | **29** |
|  |  | 7.6 | -6.8E-05 | 5.8E-09 | - | 0.42 | 31 |
|  |  | 7.6 | 7.8E-05 | -1.4E-07 | 4.4E-11 | 0.44 | 32 |
|  | Temp | **7.5** | **5.3E-03** | **-** | **-** | **0.25** | **35** |
|  |  | 7.4 | 1.9E-02 | -5.5E-04 | - | 0.29 | 36 |
|  |  | 7.4 | 1.9E-02 | -5.1E-04 | -1.1E-06 | 0.29 | 38 |
| (2) species richness of  “not-anomalous” sites | Prec | **2.7** | **-1.2E-03** | **-** | **-** | **0.43** | **1289** |
|  |  | 2.9 | -1.8E-03 | 3.0E-07 | - | 0.43 | 1280 |
|  |  | 2.7 | -7.7E-04 | -8.1E-07 | 3.6E-10 | 0.43 | 1278 |
|  | Temp | 0.4 | 1.2E-01 | - | - | 0.34 | 1488 |
|  |  | -2.7 | 6.1E-01 | -1.9E-02 | - | 0.41 | 1339 |
|  |  | **6.1** | **-1.6** | **1.6E-01** | **-4.7E-03** | **0.45** | **1249** |

**Table S4**

**List of the selected woody species from the third Spanish National Forest Inventory, using the nomenclature of the Atlas Florae Europaeae, including the species name, family, and rank (i.e. information at species or genus level).**

| **Species name** | **Family** | **Genus** | **Species** | **Rank** |
| --- | --- | --- | --- | --- |
| *Abies alba* Mill. | *Pinaceae* | *Abies* | *alba* | species |
| *Abies pinsapo* Boiss. | *Pinaceae* | *Abies* | *pinsapo* | species |
| *Acacia dealbata* Link | *Leguminosae* | *Acacia* | *dealbata* | species |
| *Acacia* sp. | *Leguminosae* | *Acacia* |  | genus |
| *Acer campestre*L. | *Aceraceae* | *Acer* | *campestre* | species |
| *Acer monspessulanum* L. | *Aceraceae* | *Acer* | *monspessulanum* | species |
| *Acer opalus* Mill. | *Aceraceae* | *Acer* | *opalus* | species |
| *Acer platanoides* L. | *Aceraceae* | *Acer* | *platanoides* | species |
| *Acer pseudoplatanus* L. | *Aceraceae* | *Acer* | *pseudoplatanus* | species |
| *Adenocarpus* sp. | *Leguminosae* | *Adenocarpus* |  | genus |
| *Alnus glutinosa* (L.) Gaertn. | *Betulaceae* | *Alnus* | *glutinosa* | species |
| *Anthyllis cytisoides*L. | *Leguminosae* | *Anthyllis* | *cytisoides* | species |
| *Arbutus unedo* L. | *Ericaceae* | *Arbutus* | *unedo* | species |
| *Arctostaphylos uva-ursi* Spreng. | *Ericaceae* | *Arctostaphylos* | *uva-ursi* | species |
| *Artemisia* sp. | *Compositae* | *Artemisia* |  | genus |
| *Asparagus* sp. | *Liliaceae* | *Asparagus* |  | genus |
| *Astragalus* sp. | *Leguminosae* | *Astragalus* |  | genus |
| *Atriplex* sp. | *Chenopodiaceae* | *Atriplex* |  | genus |
| *Berberis vulgaris* L. | *Berberidaceae* | *Berberis* | *vulgaris* | species |
| *Betula alba* L. | *Betulaceae* | *Betula* | *alba* | species |
| *Betula pendula* Roth | *Betulaceae* | *Betula* | *pendula* | species |
| *Betula* sp. | *Betulaceae* | *Betula* |  | genus |
| *Bupleurum fruticescens* L. | *Umbelliferae* | *Bupleurum* | *fruticescens* | species |
| *Bupleurum fruticosum* L. | *Umbelliferae* | *Bupleurum* | *fruticosum* | species |
| *Bupleurum* sp. | *Umbelliferae* | *Bupleurum* |  | genus |
| *Buxus sempervirens* L. | *Buxaceae* | *Buxus* | *sempervirens* | species |
| *Calicotome spinosa* (L.) Link | *Leguminosae* | *Calicotome* | *spinosa* | species |
| *Calluna vulgaris* (L.) Hull | *Ericaceae* | *Calluna* | *vulgaris* | species |
| *Castanea sativa* Mill. | *Fagaceae* | *Castanea* | *sativa* | species |
| *Celtis australis* L. | *Ulmaceae* | *Celtis* | *australis* | species |
| *Ceratonia siliqua* L. | *Leguminosae* | *Ceratonia* | *siliqua* | species |
| *Chamaecyparis lawsoniana* (A.Murray bis) Parl. | *Cupressaceae* | *Chamaecyparis* | *lawsoniana* | species |
| *Chamaespartium tridentatum* (L.) P.E.Gibbs | *Leguminosae* | *Chamaespartium* | *tridentatum* | species |
| *Cistus albidus* L. | *Cistaceae* | *Cistus* | *albidus* | species |
| *Cistus clusii* Dunal | *Cistaceae* | *Cistus* | *clusii* | species |
| *Cistus crispus* L. | *Cistaceae* | *Cistus* | *crispus* | species |
| *Cistus ladanifer* L. | *Cistaceae* | *Cistus* | *ladanifer* | species |
| *Cistus laurifolius* L. | *Cistaceae* | *Cistus* | *laurifolius* | species |
| *Cistus monspeliensis* L. | *Cistaceae* | *Cistus* | *monspeliensis* | species |
| *Cistus populifolius* L. | *Cistaceae* | *Cistus* | *populifolius* | species |
| *Cistus salvifolius* L. | *Cistaceae* | *Cistus* | *salvifolius* | species |
| *Cistus* sp. | *Cistaceae* | *Cistus* |  | genus |
| *Clematis flammula* L. | *Ranunculaceae* | *Clematis* | *flammula* | species |
| *Clematis* sp. | *Ranunculaceae* | *Clematis* |  | genus |
| *Clematis vitalba* L. | *Ranunculaceae* | *Clematis* | *vitalba* | species |
| *Colutea arborescens* L. | *Leguminosae* | *Colutea* | *arborescens* | species |
| *Corema album* (L.) D.Don | *Empetraceae* | *Corema* | *album* | species |
| *Coriaria myrtifolia* L. | *Coriariaceae* | *Coriaria* | *myrtifolia* | species |
| Coronilla emerus L. | *Leguminosae* | *Coronilla* | *emerus* | species |
| *Coronilla glauca* L. | *Leguminosae* | *Coronilla* | *glauca* | species |
| *Coronilla* sp. | *Leguminosae* | *Coronilla* |  | genus |
| *Corylus avellana* L. | *Betulaceae* | *Corylus* | *avellana* | species |
| *Cotoneaster* sp. | *Rosaceae* | *Cotoneaster* |  | genus |
| *Crataegus monogyna* Jacq. | *Rosaceae* | *Crataegus* | *monogyna* | species |
| *Crataegus* sp. | *Rosaceae* | *Crataegus* |  | genus |
| *Cytisophyllum sessilifolium* (L.) O.Lang | *Leguminosae* | *Cytisophyllum* | *sessilifolium* | species |
| *Cytisus* sp. | *Leguminosae* | *Cytisus* |  | genus |
| *Daboecia cantabrica* (Huds.) K.Koch | *Ericaceae* | *Daboecia* | *cantabrica* | species |
| *Daphne gnidium* L. | *Thymelaeaceae* | *Daphne* | *gnidium* | species |
| *Daphne laureola* L. | *Thymelaeaceae* | *Daphne* | *laureola* | species |
| *Daphne mezereum* L. | *Thymelaeaceae* | *Daphne* | *mezereum* | species |
| *Daphne* sp. | *Thymelaeaceae* | *Daphne* |  | genus |
| *Dorycnium hirsutum* (L.) Ser. | *Leguminosae* | *Dorycnium* | *hirsutum* | species |
| *Dorycnium pentaphyllum* Scop. | *Leguminosae* | *Dorycnium* | *pentaphyllum* | species |
| *Dorycnium* sp. | *Leguminosae* | *Dorycnium* |  | genus |
| *Echium* sp. | *Boraginaceae* | *Echium* |  | genus |
| *Erica arborea* L. | *Ericaceae* | *Erica* | *arborea* | species |
| *Erica australis* L. | *Ericaceae* | *Erica* | *australis* | species |
| *Erica cinerea* L. | *Ericaceae* | *Erica* | *cinerea* | species |
| *Erica multiflora* L. | *Ericaceae* | *Erica* | *multiflora* | species |
| *Erica scoparia* L. | *Ericaceae* | *Erica* | *scoparia* | species |
| *Erica* sp. | *Ericaceae* | *Erica* |  | genus |
| *Erica vagans* L. | *Ericaceae* | *Erica* | *vagans* | species |
| *Erinacea anthyllis* Link | *Leguminosae* | *Erinacea* | *anthyllis* | species |
| *Erinacea* sp. | *Leguminosae* | *Erinacea* |  | genus |
| *Eucalyptus camaldulensis* Dehnh. | *Myrtaceae* | *Eucalyptus* | *camaldulensis* | species |
| *Eucalyptus globulus* Labill. | *Myrtaceae* | *Eucalyptus* | *globulus* | species |
| *Eucalyptus nitens* (H.Deane & Maiden) Maiden | *Myrtaceae* | *Eucalyptus* | *nitens* | species |
| *Euphorbia* sp. | *Euphorbiaceae* | *Euphorbia* |  | genus |
| *Fagus sylvatica* L. | *Fagaceae* | *Fagus* | *sylvatica* | species |
| *Ficus carica* L. | *Moraceae* | *Ficus* | *carica* | species |
| *Frangula alnus* Mill. | *Rhamnaceae* | *Frangula* | *alnus* | species |
| *Fraxinus angustifolia* Vahl | *Oleaceae* | *Fraxinus* | *angustifolia* | species |
| *Fraxinus excelsior* L. | *Oleaceae* | *Fraxinus* | *excelsior* | species |
| *Genista balansae* Rouy | *Leguminosae* | *Genista* | *balansae* | species |
| *Genista monspessulana* (L.) L.A.S. Johnson | *Leguminosae* | *Genista* | *monspessulana* | species |
| *Genista patens* (L. ex Murr.) | *Leguminosae* | *Genista* | *patens* | species |
| *Genista scorpius* (L.) DC. | *Leguminoseae* | *Genista* | *scorpius* | species |
| *Genista* sp. | *Leguminosae* | *Genista* |  | genus |
| *Genista triflora* (L’Hérit.) Rouy | *Leguminosae* | *Genista* | *triflora* | species |
| *Genista umbellata* (L'Hér.) Poir. | *Leguminoseae* | *Genista* | *umbellata* | species |
| *Genistella* sp. | *Leguminosae* | *Genistella* |  | genus |
| *Globularia alypum* L. | *Globulariaceae* | *Globularia* | *alypum* | species |
| *Halimium halimifolium* (L.) Willk. | *Cistaceae* | *Halimium* | *halimifolium* | species |
| *Halimium* sp. | *Cistaceae* | *Halimium* |  | genus |
| *Hedera helix* L. | *Araliaceae* | *Hedera* | *helix* | species |
| *Helianthemum* sp. | *Cistaceae* | *Helianthemum* |  | genus |
| *Helichrysum stoechas* (L.) Moench | *Compositae* | *Helichrysum* | *stoechas* | species |
| *Ilex aquifolium* L. | *Aquifoliaceae* | *Ilex* | *aquifolium* | species |
| *Jasminum fruticans* L. | *Oleaceae* | *Jasminum* | *fruticans* | species |
| *Juglans regia* L. | *Juglandaceae* | *Juglans* | *regia* | species |
| *Juniperus communis* L. | *Cupressaceae* | *Juniperus* | *communis* | species |
| *Juniperus oxycedrus* L. | *Cupressaceae* | *Juniperus* | *oxycedrus* | species |
| *Juniperus phoenicea* L. | *Cupressaceae* | *Juniperus* | *phoenicea* | species |
| *Juniperus sabina* L. | *Cupressaceae* | *Juniperus* | *sabina* | species |
| *Juniperus thurifera* L. | *Cupressaceae* | *Juniperus* | *thurifera* | species |
| *Larix* sp. | *Pinaceae* | *Larix* |  | genus |
| *Laurus nobilis* L. | *Lauraceae* | *Laurus* | *nobilis* | species |
| *Lavandula lanata* Boiss. | *Labiatae* | *Lavandula* | *lanata* | species |
| *Lavandula latifolia* Medik. | *Labiatae* | *Lavandula* | *latifolia* | species |
| *Lavandula* sp. | *Labiatae* | *Lavandula* |  | genus |
| *Lavandula stoechas* L. | *Labiatae* | *Lavandula* | *stoechas* | species |
| *Ligustrum vulgare* L. | *Oleaceae* | *Ligustrum* | *vulgare* | species |
| *Lonicera etrusca* Santi | *Caprifoliaceae* | *Lonicera* | *etrusca* | species |
| *Lonicera implexa* Aiton | *Caprifoliaceae* | *Lonicera* | *implexa* | species |
| *Lonicera periclymenum* L. | *Caprifoliaceae* | *Lonicera* | *periclymenum* | species |
| *Lonicera pyrenaica* L. | *Caprifoliaceae* | *Lonicera* | *pyrenaica* | species |
| *Lonicera* sp. | *Caprifoliaceae* | *Lonicera* |  | genus |
| *Lonicera xylosteum* L. | *Caprifoliaceae* | *Lonicera* | *xylosteum* | species |
| *Malus sylvestris* Mill. | *Rosaceae* | *Malus* | *sylvestris* | species |
| *Nerium oleander* L. | *Apocynaceae* | *Nerium* | *oleander* | species |
| *Olea europaea* L. | *Oleaceae* | *Olea* | *europaea* | species |
| *Ononis* sp. | *Leguminosae* | *Ononis* |  | genus |
| *Ononis tridentata* L. | *Leguminosae* | *Ononis* | *tridentata* | species |
| *Osyris* sp. | *Santalaceae* | *Osyris* |  | genus |
| *Phillyrea angustifolia* L. | *Oleaceae* | *Phillyrea* | *angustifolia* | species |
| *Phillyrea latifolia* L. | *Oleaceae* | *Phillyrea* | *latifolia* | species |
| *Phlomis lychnitis* L. | *Labiatae* | *Phlomis* | *lychnitis* | species |
| *Phlomis purpurea* L. | *Labiatae* | *Phlomis* | *purpurea* | species |
| *Phlomis* sp. | *Labiatae* | *Phlomis* |  | genus |
| *Picea abies* (L.) H.Karst. | *Pinaceae* | *Picea* | *abies* | species |
| *Pinus halepensis* Mill. | *Pinaceae* | *Pinus* | *halepensis* | species |
| *Pinus nigra* J.F.Arnold | *Pinaceae* | *Pinus* | *nigra* | species |
| *Pinus pinaster* Aiton | *Pinaceae* | *Pinus* | *pinaster* | species |
| *Pinus pinea* L. | *Pinaceae* | *Pinus* | *pinea* | species |
| *Pinus radiata*D.Don | *Pinaceae* | *Pinus* | *radiata* | species |
| *Pinus sylvestris* L. | *Pinaceae* | *Pinus* | *sylvestris* | species |
| *Pinus uncinata* Mill. ex Mirb. | *Pinaceae* | *Pinus* | *uncinata* | species |
| *Pistacia lentiscus* L. | *Anacardiaceae* | *Pistacia* | *lentiscus* | species |
| *Pistacia terebinthus* L. | *Anacardiaceae* | *Pistacia* | *terebinthus* | species |
| *Platanus hispanica* Ten. | *Platanaceae* | *Platanus* | *hispanica* | species |
| *Populus alba*L. | *Salicaceae* | *Populus* | *alba* | species |
| *Populus nigra* L. | *Salicaceae* | *Populus* | *nigra* | species |
| *Populus tremula* L. | *Salicaceae* | *Populus* | *tremula* | species |
| *Populus x canadensis* Moench | *Salicaceae* | *Populus* | *x canadensis* | species |
| *Prunus avium* L. | *Rosaceae* | *Prunus* | *avium* | species |
| *Prunus mahaleb* L. | *Rosaceae* | *Prunus* | *mahaleb* | species |
| *Prunus* sp. | *Rosaceae* | *Prunus* |  | genus |
| *Pseudotsuga menziesii* (Mirb.) Franco | *Pinaceae* | *Pseudotsuga* | *menziesii* | species |
| *Pyrus* sp. | *Rosaceae* | *Pyrus* |  | genus |
| *Quercus canariensis* Willd. | *Fagaceae* | *Quercus* | *canariensis* | species |
| *Quercus coccifera* L. | *Fagaceae* | *Quercus* | *coccifera* | species |
| *Quercus faginea* Lam. | *Fagaceae* | *Quercus* | *faginea* | species |
| *Quercus fruticosa* Brot. | *Fagaceae* | *Quercus* | *fruticosa* | species |
| *Quercus ilex* L. | *Fagaceae* | *Quercus* | *ilex* | species |
| *Quercus petraea* (Matt.) Liebl. | *Fagaceae* | *Quercus* | *petraea* | species |
| *Quercus pubescens* Willd. (Q. Humilis) | *Fagaceae* | *Quercus* | *pubescens* | species |
| *Quercus pyrenaica* Willd. | *Fagaceae* | *Quercus* | *pyrenaica* | species |
| *Quercus robur* L. | *Fagaceae* | *Quercus* | *robur* | species |
| *Quercus rubra* L. | *Fagaceae* | *Quercus* | *rubra* | species |
| *Quercus suber* L. | *Fagaceae* | *Quercus* | *suber* | species |
| *Retama sphaerocarpa* (L.) Boiss. | *Leguminosae* | *Retama* | *sphaerocarpa* | species |
| *Retama* sp. | *Leguminosae* | *Retama* |  | genus |
| *Rhamnus alaternus*L. | *Rhamnaceae* | *Rhamnus* | *alaternus* | species |
| *Rhamnus alpinus* L. | *Rhamnaceae* | *Rhamnus* | *alpinus* | species |
| *Rhamnus lycioides* L. | *Rhamnaceae* | *Rhamnus* | *lycioides* | species |
| *Rhamnus myrtifolius* Willk. | *Rhamnaceae* | *Rhamnus* | *myrtifolius* | species |
| *Rhamnus oleoides* L. | *Rhamnaceae* | *Rhamnus* | *myrtifolius* | species |
| *Rhamnus saxatilis* Jacq. | *Rhamnaceae* | *Rhamnus* | *saxatilis* | species |
| *Rhamnus* sp. | *Rhamnaceae* | *Rhamnus* |  | genus |
| *Rhododendron* sp. | *Ericaceae* | *Rhododendron* |  | genus |
| *Ribes alpinum* L. | *Grossulariaceae* | *Ribes* | *alpinum* | species |
| *Ribes rubrum* L. | *Grossulariaceae* | *Ribes* | *rubrum* | species |
| *Ribes* sp. | *Grossulariaceae* | *Ribes* |  | genus |
| *Robinia pseudacacia* L. | *Leguminosae* | *Robinia* | *pseudacacia* | species |
| *Rosa* sp. | *Rosaceae* | *Rosa* |  | genus |
| *Rosmarinus officinalis* L. | *Labiatae* | *Rosmarinus* | *officinalis* | species |
| *Rubus caesius* L. | *Rosaceae* | *Rubus* | *caesius* | species |
| *Rubus idaeus* L. | *Rosaceae* | *Rubus* | *idaeus* | species |
| *Rubus* sp. | *Rosaceae* | *Rubus* |  | genus |
| *Rubus ulmifolius* Schott | *Rosaceae* | *Rubus* | *ulmifolius* | species |
| *Ruscus aculeatus* L. | *Liliaceae* | *Ruscus* | *aculeatus* | species |
| *Salix* sp. | *Salicaceae* | *Salix* |  | genus |
| *Sambucus nigra* L. | *Caprifoliaceae* | *Sambucus* | *nigra* | species |
| *Santolina rosmarinifolia*L. | *Compositae* | *Santolina* | *rosmarinifolia* | species |
| *Sarothamnus scoparius* L. | *Fagaceae* | *Sarothamnus* | *scoparius* | species |
| *Sarothamnus vulgaris*Wimm. | *Fagaceae* | *Sarothamnus* | *vulgaris* | species |
| *Smilax aspera* L. | *Liliaceae* | *Smilax* | *aspera* | species |
| *Sorbus aria* (L.) Crantz | *Rosaceae* | *Sorbus* | *aria* | species |
| *Sorbus aucuparia* L. | *Rosaceae* | *Sorbus* | *aucuparia* | species |
| *Sorbus* sp. | *Rosaceae* | *Sorbus* |  | genus |
| *Sorbus torminalis* (L.) Crantz | *Rosaceae* | *Sorbus* | *torminalis* | species |
| *Spartium junceum* L. | *Leguminosae* | *Spartium* | *junceum* | species |
| *Spartium* sp. | *Leguminosae* | *Spartium* |  | genus |
| *Spiraea* sp. | *Rosaceae* | *Spiraea* |  | genus |
| *Tamarix* sp. | *Tamaricaceae* | *Tamarix* |  | genus |
| *Taxus baccata* L. | *Taxaceae* | *Taxus* | *baccata* | species |
| *Teline* sp. | *Leguminosae* | *Teline* |  | genus |
| *Thymelaea* sp. | *Thymelaeaceae* | *Thymelaea* |  | genus |
| *Thymus mastichina* L. | *Labiatae* | *Thymus* | *mastichina* | species |
| *Thymus* sp. | *Labiatae* | *Thymus* |  | genus |
| *Tilia cordata* Mill. | *Tiliaceae* | *Tilia* | *cordata* | species |
| *Tilia platyphyllos* Scop. | *Tiliaceae* | *Tilia* | *platyphyllos* | species |
| *Tilia* sp. | *Tiliaceae* | *Tilia* |  | genus |
| *Ulex parviflorus* Pourr. | *Leguminosae* | *Ulex* | *parviflorus* | species |
| *Ulex* sp. | *Leguminosae* | *Ulex* |  | genus |
| *Ulmus glabra* Huds. | *Ulmaceae* | *Ulmus* | *glabra* | species |
| *Ulmus minor* Mill. | *Ulmaceae* | *Ulmus* | *minor* | species |
| *Vaccinium myrtillus* L. | *Ericaceae* | *Vaccinium* | *myrtillus* | species |
| *Viburnum rigidum* Vent. | *Caprifoliaceae* | *Viburnum* | *rigidum* | species |
| *Viburnum* sp. | *Caprifoliaceae* | *Viburnum* |  | genus |
